# Cytogenetic Evidence for Sex Chromosomes and Karyotype Evolution in Anguimorphan Lizards

**DOI:** 10.3390/cells10071612

**Published:** 2021-06-28

**Authors:** Barbora Augstenová, Eleonora Pensabene, Lukáš Kratochvíl, Michail Rovatsos

**Affiliations:** Department of Ecology, Faculty of Science, Charles University, 12844 Prague, Czech Republic; augstenova.barbora@gmail.com (B.A.); pensabee@natur.cuni.cz (E.P.); kratoch1@natur.cuni.cz (L.K.)

**Keywords:** evolution, CGH, FISH, karyotype, rDNA, sex chromosomes, telomeres

## Abstract

Anguimorphan lizards are a morphologically variable group of squamate reptiles with a wide geographical distribution. In spite of their importance, they have been cytogenetically understudied. Here, we present the results of the cytogenetic examination of 23 species from five anguimorphan families (Anguidae, Helodermatidae, Shinisauridae, Varanidae and Xenosauridae). We applied both conventional (Giemsa staining and C-banding) and molecular cytogenetic methods (fluorescence *in situ* hybridization with probes for the telomeric motifs and rDNA loci, comparative genome hybridization), intending to describe the karyotypes of previously unstudied species, to uncover the sex determination mode, and to reveal the distribution of variability in cytogenetic characteristics among anguimorphan lizards. We documented that karyotypes are generally quite variable across anguimorphan lineages, with anguids being the most varying. However, the derived chromosome number of 2n = 40 exhibits a notable long-term evolutionary stasis in monitors. Differentiated ZZ/ZW sex chromosomes were documented in monitors and helodermatids, as well as in the anguids *Abronia lythrochila*, and preliminary also in *Celestus warreni* and *Gerrhonotus liocephalus*. Several other anguimorphan species have likely poorly differentiated sex chromosomes, which cannot be detected by the applied cytogenetic methods, although the presence of environmental sex determination cannot be excluded. In addition, we uncovered a rare case of spontaneous triploidy in a fully grown *Varanus primordius*.

## 1. Introduction

Sex chromosomes have evolved independently across vertebrates multiple times [1,2,3]. They mostly evolve from a pair of autosomes by the emergence of a sex-determining gene, and may further differentiate via the suppression of recombination leading to the loss of functional genes, accumulation of heterochromatin and/or repetitive elements in the sex-specific chromosome [4,5,6]. For decades, conventional and, more recently, molecular cytogenetic methods have been used to identify sex chromosomes and the degree of their differentiation. Cytogenetic analysis showed that the process of sex chromosome differentiation varies significantly among lineages: some lineages such as skinks possessed for a long evolutionary time relatively poorly differentiated, cytogenetically hardly distinguishable sex chromosomes [7], while others such as the chameleons of the genus *Furcifer* evolved heteromorphic sex chromosomes in a much shorter evolutionary time than, e.g., skinks [8,9]. Sex chromosomes are often detectable by cytogenetic techniques due to their unusual content of repetitive sequences. Extensive accumulation of various repetitive elements such as rDNA loci, microsatellite motifs, and retrotransposons has been detected in sex chromosomes of many animal and plant species, often in heterochromatic regions [10,11,12,13,14,15,16,17,18,19].

Anguimorpha is a group of squamate reptiles distributed in all continents except Antarctica, containing around 240 extant species [20] divided into seven families: Anguidae, Anniellidae, Helodermatidae, Lanthanotidae, Shinisauridae, Varanidae, and Xenosauridae [21]. The most species-rich anguimorphan groups are the families Anguidae (slow worms, glass lizards, and alligator lizards) and Varanidae (monitors) [20]. Monitors are also the cytogenetically most studied group of the anguimorphan clade. The karyotype has been so far described in 30 species [16,22,23,24,25,26,27,28,29,30,31,32,33]. All species of monitors show karyotypes with the 2n = 40 chromosomes (16 macro- and 24 microchromosomes) and similar morphology of chromosomes. A notable variability has been detected in them only in the morphology of the chromosome pairs 5–8, which can be attributed to intrachromosomal rearrangements according to the results of chromosome painting [33]. All studied species of monitors show ZZ/ZW system of sex determination [33,34]. The sex chromosomes were cytogenetically identified in several species of monitors as a pair of microchromosomes, where the W is usually heterochromatic [16,25,33]. Comparative chromosome painting [33] and comparison of the differences in gene copy numbers of Z-specific genes between sexes by quantitative PCR (qPCR) revealed an extensive homology of the sex chromosomes across monitors [34]. The Z chromosome of monitors is largely homologous to chicken chromosome 28, and the gene *anti-Müllerian hormone* (*amh)* was proposed as a candidate sex-determining locus [34,35].

In addition to monitors, sex chromosomes were uncovered by cytogenetic methods in *Heloderma suspectum* (Helodermatidae) [36]. The sex chromosomes of this species are microchromosomes with highly heterochromatic W [36]. The qPCR test of sex chromosome homology revealed that three tested species of helodermatids (*Heloderma suspectum, Heloderma horridum* and *Heloderma exasperatum*) as well as the alligator lizard *Abronia lythrochila* (Anguidae) share the same sex chromosomes with monitors [34]. The homology of the Z chromosomes among the families Anguidae, Helodermatidae, and Varanidae suggests that the age of the anguimorphan sex chromosomes can be estimated to 115–180 MY [34]. Interestingly, the tested Z-specific genes of monitors and helodermatids were pseudoautosomal or autosomal in the slow worm *Anguis fragilis* (Anguidae) suggesting that the sex chromosomes of certain species of anguids might not be homologous to those of the other studied anguimorphan lizards [34].

Previous studies described the karyotypes of one unspecified species of the genus *Xenosaurus* (Xenosauridae), three species of the family Anniellidae (*Anniella geronimensis, Anniella pulchra, Anniella stebbinsi*) and 12 species of the family Anguidae (*Abronia monticola, Anguis fragilis, Anguis veronensis, Celestus costatus, Diploglossus fasciatus, Diploglossus millepunctatus, Elgaria coerulea, Elgaria multicarinata, Elgaria paucicarinata, Ophiodes striatus, Ophisaurus ventralis, Pseudopus apodus* [37,38,39,40,41,42,43]. Sex chromosomes were not identified in any of these species; however, they might escape detection, as in some cases individuals of single or unknown sex were studied and mostly only conventional cytogenetic methods were applied.

Our current knowledge of the sex determination systems in anguimorphan lizards has been limited to monitors, helodermatids, and a single species of alligator lizard [33,34,36]. In the current study, we examined anguimorphan lizards from five families (Anguidae, Helodermatidae, Shinisauridae, Xenosauridae, and Varanidae) by both conventional (Giemsa staining, C-banding) and molecular cytogenetic methods (fluorescence *in situ* hybridization with probes for telomeric motifs and rDNA loci, comparative genome hybridization).

## 2. Materials and Methods

### 2.1. Studied Material, Chromosome Preparations and Staining

We studied 23 species from five families of Anguimorpha: Abronia campbelli, Abronia deppii, Abronia graminea, Abronia lythrochila, Abronia mixteca, Abronia smithi, Abronia taeniata, Barisia rudicollis, Celestus warreni, Gerrhonotus liocephalus (Anguidae), Heloderma exasperatum, Heloderma horridum (Helodermatidae), Shinisaurus crocodilurus (Shinisauridae), Varanus auffenbergi, Varanus cumingi, Varanus kordensis, Varanus olivaceus, Varanus primordius, Varanus salvadorii, Varanus salvator komaini (Varanidae), Xenosaurus grandis, Xenosaurus platyceps, Xenosaurus rectocollaris (Xenosauridae) (Table S1).

We collected 0.2–2 mL of peripheral blood, which was subsequently used for DNA isolation and preparation of mitotic chromosome suspensions. DNA was isolated using the DNeasy Blood and Tissue Kit (Qiagen, Valencia, CA, USA) following the manufacturer’s protocol. We used whole blood cell cultures to prepare mitotic chromosome suspensions according to our standard protocol described in [44].

We used C-banding to determine the distribution of constitutive heterochromatin. Slides were treated according to the protocol of Sumner [45] with slight modifications: first, we treated the slides with 0.2 N HCl for 35 min at room temperature, then in 5% Ba(OH)_2_ for 9 min at 45 °C, and subsequently in 2× SSC for 1 h at 60 °C. In a final step, the slides were washed with distilled water, air-dried, and stained with Fluoroshield mounting medium with DAPI (4′, 6-diamidino-2-phenylindole; Sigma-Aldrich, St. Louis, MO, USA).

### 2.2. Fluorescence *In Situ* Hybridization with Probe for Telomeric Sequences

For the Fluorescence *in situ* Hybridization (FISH) with telomeric probes, we used standard protocols of Ijdo et al. [46] and Mazzoleni et al. [44]. The telomeric probe (TTAGGG)_n_ was prepared by Polymerase Chain Reaction (PCR) without DNA template using the primers (TTAGGG)_5_ and (CCCTAA)_5_. The probe was precipitated overnight at −20 °C by sodium acetate (3M), salmon sperm, and ethanol and subsequently diluted in 50% formamide in 2× SSC (pH 7).

The chromosomal spreads were treated according to the protocol described in [44]. Briefly, the slides were treated with RNAse (100 μg/mL) for 60 min at 37 °C, and washed in 2× saline–sodium citrate (2× SSC) buffer. They were subsequently treated with 0.01% pepsin for 10 min at 37 °C, washed in phosphate-buffered saline (PBS), then treated with the solution of 1% formaldehyde in 2× SSC, and afterwards washed again in PBS. Slides were dehydrated in ethanol series and air-dried. Dried slides were denatured in 70% formamide in 2× SSC at 70 °C for 3 min, washed in 2× SSC, and again dehydrated in ethanol series and air-dried. The probe was applied to the chromosome spreads for overnight hybridization at 37 °C.

The following day the slides were washed three times in 50% formamide in 2× SSC at 37 °C for 5 min, then in 2× SSC and subsequently in 4× SSC/0.05% Tween20 (Sigma-Aldrich) for 5 min. Afterwards, we applied 4× SSC/5% blocking reagent (Roche, Basel, Switzerland) and incubated the slides for 45 min at 37 °C. The slides were washed in 4× SSC/0.05% Tween20 and incubated with 4× SSC/5% blocking reagent with avidin-FITC (Vector Laboratories, Burlingame, CA, USA) for 30 min at 37 °C. The signal was amplified by using avidin-FITC/biotinylated anti-avidin system (Vector Laboratories Burlingame, CA, USA). Subsequently, the slides were washed in 4× SSC/0.05% Tween20 and PBS solution, dehydrated in ethanol series and air-dried. The slides were stained by Fluoroshield mounting medium with DAPI (Sigma-Aldrich, St. Louis, MO, USA).

### 2.3. Fluorescence *In Situ* Hybridization with Probe for 18S/28S rDNA Loci

A plasmid (pDmr.a 51#1) with an 11.5-kb insert encoding the 18S and 28S ribosomal units of *Drosophila melanogaster* [47] was used to prepare the probe for the rDNA loci, according to the protocol of Rovatsos et al. [8]. The probe was labeled with dUTP-biotin (Roche, Basel, Switzerland) by nick translation using the manufacturer’s protocol of Nick Translation Kit (Abbott Laboratories, Lake Bluff, IL, USA). The probe was precipitated and the FISH was performed according to the protocol described above.

### 2.4. Comparative Genome Hybridization

For comparative genome hybridization (CGH) we used a standard protocol described in Rovatsos et al. [9]. The probes were labeled by the Nick Translation Kit (Abbott Laboratories, Lake Bluff, IL, USA) according to the manufacturer’s protocol. The female-specific probe was labeled by dUTP-digoxigenin (Roche, Basel, Switzerland) and the male-specific probe by dUTP-biotin (Roche, Basel, Switzerland). We prepared the probes for both male and female genome per species with equal concentrations and mixed them during the probe precipitation. The probes were precipitated as described above. Chromosome preparation and denaturation were the same as in the FISH experiments. The probes hybridized for 48 h. For the signal detection, the slides were first washed three times in 50% formamide in 2× SSC at 37 °C for 5 min, subsequently twice in 2× SSC and once in 4× SSC/0.05% Tween20 for 5 min. In the next step, we applied 4× SSC/5% blocking reagent (Roche, Basel, Switzerland) for 30 min at 37 °C. Afterwards, we incubated the slides with 4× SSC/5% blocking reagent with avidin-FITC (Vector Laboratories Burlingame, CA, USA) and anti-digoxigenin-Rhodamine (Roche, Basel, Switzerland) for 30 min at 37 °C. In the end, the slides were washed twice in 4× SSC/0.05% Tween20, once in PBS solution, and dehydrated in ethanol series. The slides were air-dried and stained by Fluoroshield with DAPI (Sigma-Aldrich, St. Louis, MO, USA).

### 2.5. Microscopy and Image Analyses

The Giemsa-stained slides were scanned with an Axio Imager Z2 microscope (Zeiss, Oberkochen, Germany) equipped with Metafer-MSearch scanning platform and a CoolCube 1 b/w digital camera (MetaSystems, Altlussheim, Germany). The karyograms were constructed in the program Ikaros (MetaSystems). The photos of slides stained by DAPI (slides from C-banding and FISH experiments) were taken by a Provis AX70 microscope (Olympus, Tokyo, Japan) equipped with a DP30BW camera (Olympus). The photos were subsequently processed in DP Manager (Olympus).

### 2.6. qPCR Test for Sex Chromosome Constitution in the Triploid Varanus primordius

The cytogenetic analysis revealed a spontaneous triploid *Varanus primordius*. We applied a qPCR approach, based on Rovatsos et al. [34], to estimate the number of Z chromosomes per cell and, therefore, to reveal the sex chromosome constitution. For the qPCR test, we used primers for two autosomal genes (*adarb2*, *eef1a*, *mecom*) and one Z-specific gene (*grin3b*), designed by Rovatsos et al. [34] (Table S2).

## 3. Results

**Figure 1:**
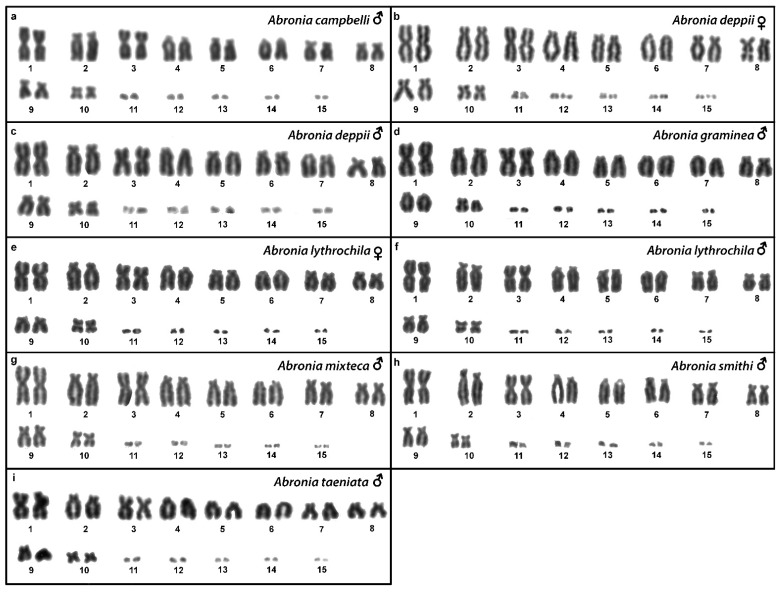
Giemsa-stained karyograms of *Abronia campbelli* (**a**), *Abronia deppii* (**b**,**c**), *Abronia graminea* (**d**), *Abronia lythrochila* (**e**,**f**), *Abronia mixteca* (**g**), *Abronia smithi* (**h**) and *Abronia taeniata* (**i**).

**Figure 2:**
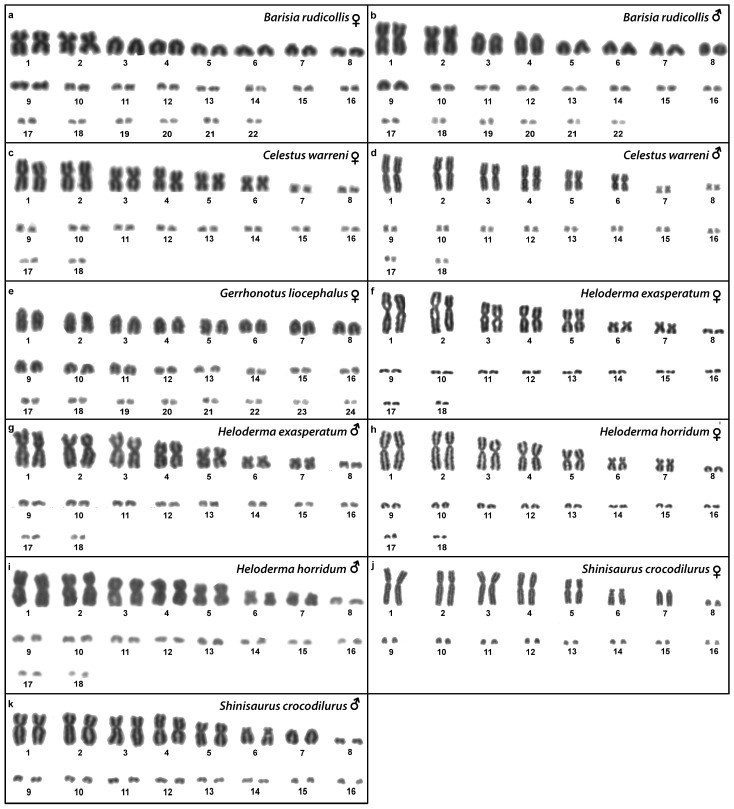
Giemsa-stained karyograms of *Barisia rudicollis* (**a**,**b**), *Celestus warreni* (**c**,**d**), *Gerrhonotus liocephalus* (**e**), *Heloderma exasperatum* (**f**,**g**), *Heloderma horridum* (**h**,**i**) and *Shinisaurus crocodilurus* (**j**,**k**).

**Figure 3:**
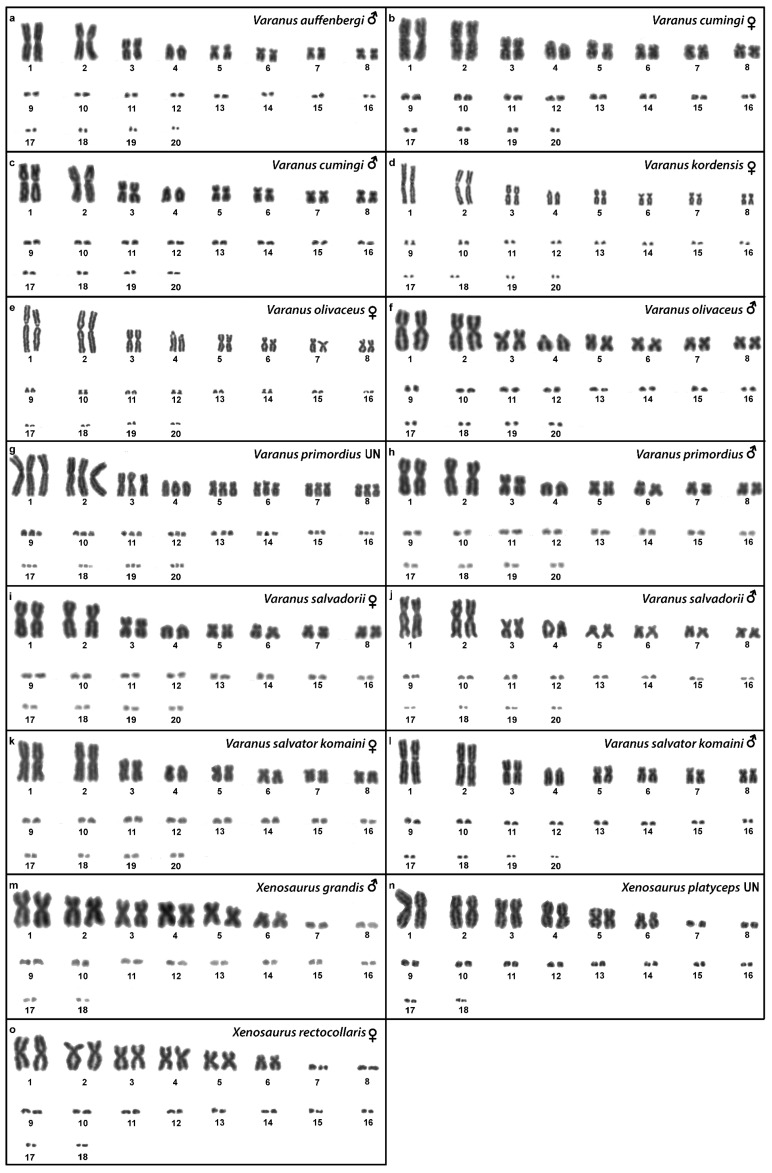
Giemsa-stained karyograms of *Varanus auffenbergi* (**a**), *Varanus cumingi* (**b**,**c**), *Varanus kordensis* (**d**), *Varanus olivaceus* (**e**,**f**), *Varanus primordius* (**g**,**h**), *Varanus salvadorii* (**i**,**j**), *Varanus salvator komaini* (**k**,**l**), *Xenosaurus grandis* (**m**), *Xenosaurus platyceps* (**n**) and *Xenosaurus rectocollaris* (**o**). UN: sex not known.

**Figure 4:**
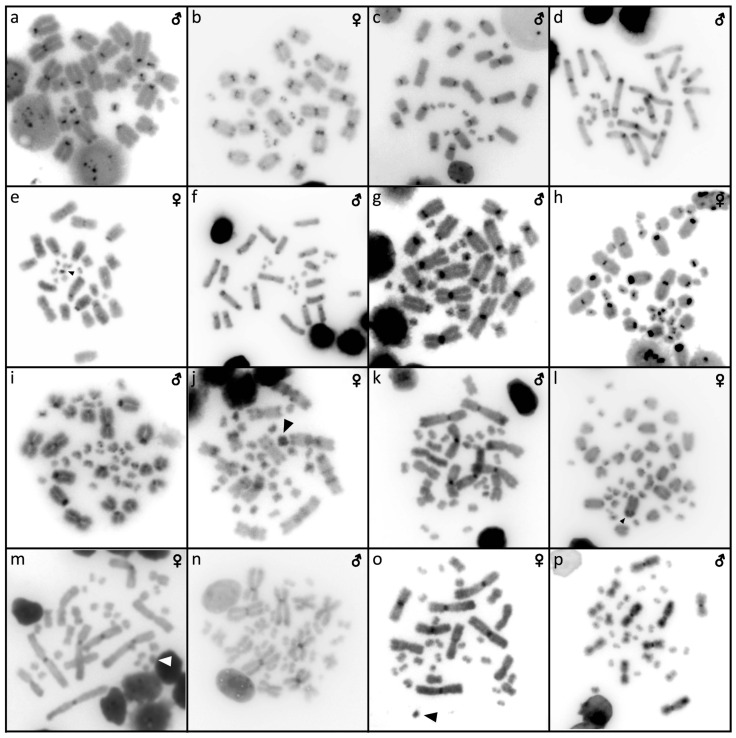
C-banded metaphases of *Abronia campbelli* (**a**), *Abronia deppii* (**b**,**c**), *Abronia graminea* (**d**), *Abronia lythrochila* (**e**,**f**), *Abronia mixteca* (**g**), *Barisia rudicollis* (**h**,**i**), *Celestus warreni* (**j**,**k**), *Gerrhonotus liocephalus* (**l**), *Heloderma exasperatum* (**m**,**n**) and *Heloderma horridum* (**o**,**p**). When visible, the W chromosomes are indicated by arrows.

**Figure 5:**
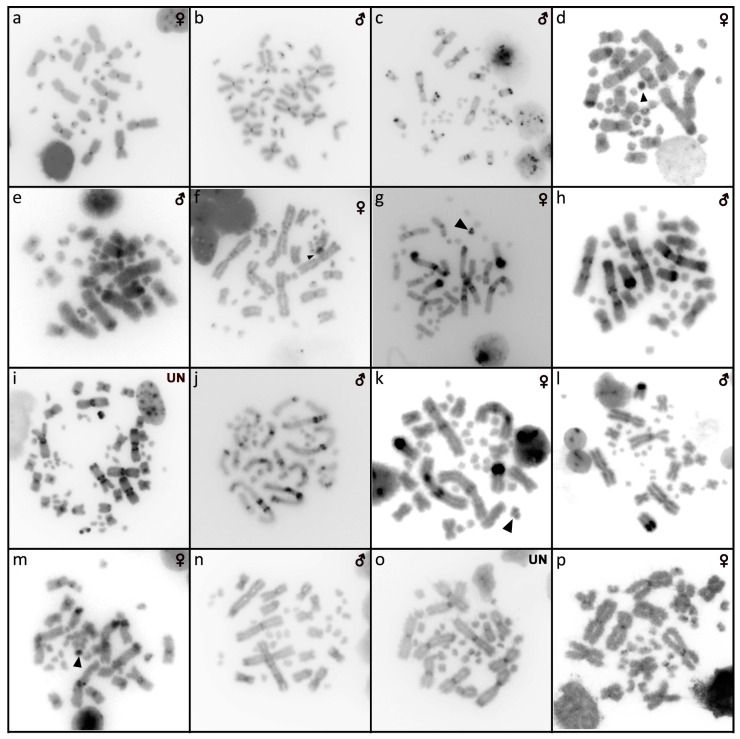
C-banded metaphases of Shinisaurus crocodilurus (**a**,**b**), *Varanus auffenbergi* (**c**), *Varanus cumingi* (**d**,**e**), *Varanus kordensis* (**f**), *Varanus olivaceus* (**g**,**h**), *Varanus primordius* (**i**,**j**), *Varanus salvadorii* (**k**,**l**), *Varanus salvator komaini* (**m**,**n**), *Xenosaurus platyceps* (**o**), and *Xenosaurus rectocollaris* (**p**). When visible, the W chromosomes are indicated by arrows. UN: sex not known.

**Figure 6:**
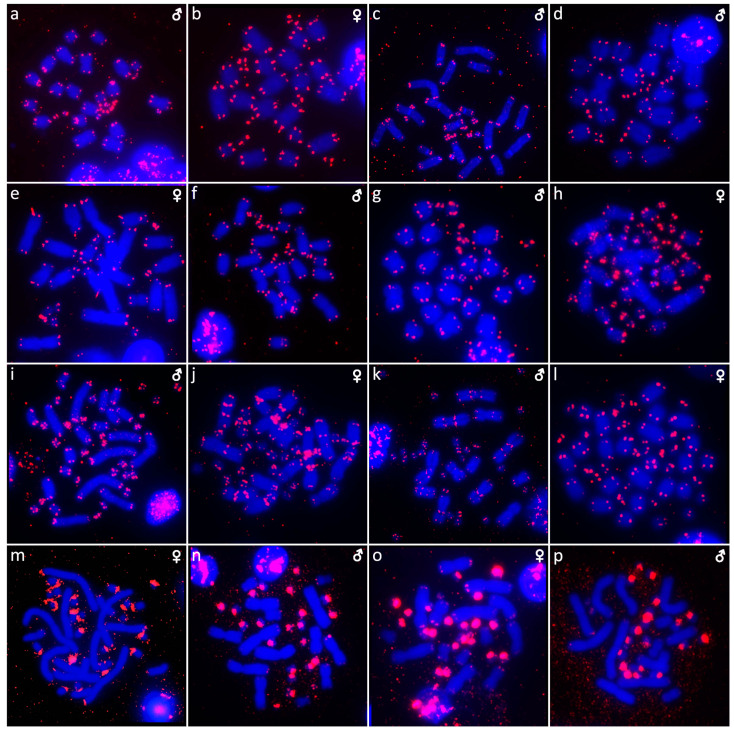
In situ hybridization with probe for telomeric sequences in *Abronia campbelli* (**a**), *Abronia deppii* (**b**,**c**), *Abronia graminea* (**d**), *Abronia lythrochila* (**e**,**f**), *Abronia mixteca* (**g**), *Barisia rudicollis* (**h**,**i**), *Celestus warreni* (**j**,**k**), *Gerrhonotus liocephalus* (**l**), *Heloderma exasperatum* (**m**,**n**) and *Heloderma horridum* (**o**,**p**).

**Figure 7:**
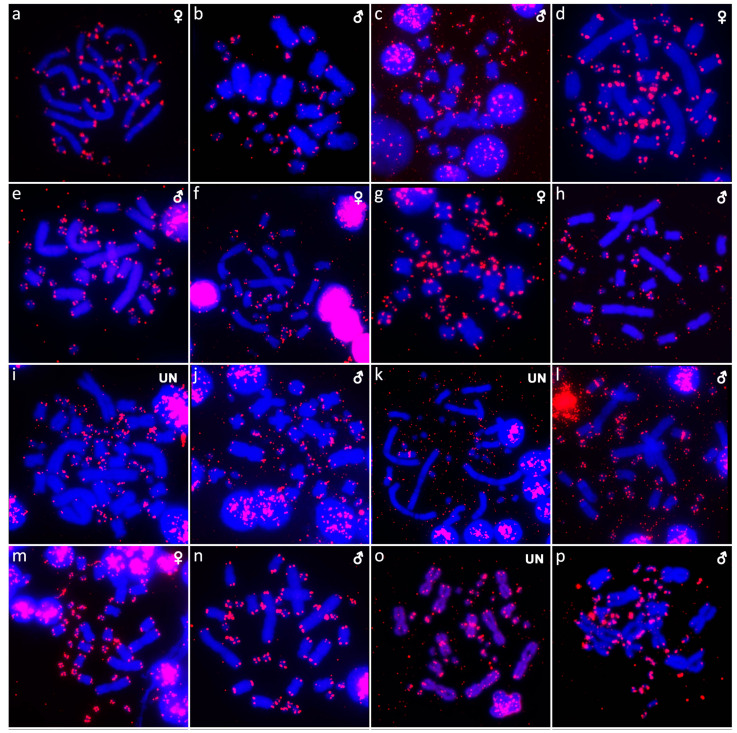
In situ hybridization with probe for telomeric sequences in *Shinisaurus crocodilurus* (**a**,**b**), *Varanus auffenbergi* (**c**), *Varanus cumingi* (**d**,**e**), *Varanus kordensis* (**f**), *Varanus primordius* (**g**,**h**), *Varanus olivaceus* (**i**,**j**), *Varanus salvadorii* (**k**,**l**), *Varanus salvator komaini* (**m**,**n**), *Xenosaurus platyceps* (**o**) and *Xenosaurus rectocollaris* (**p**). UN: sex not known.

**Figure 8:**
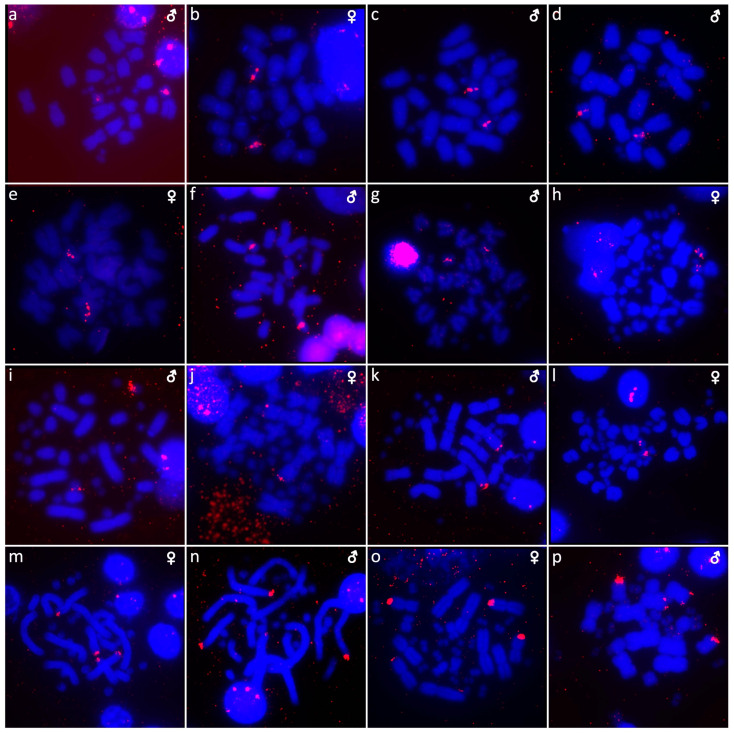
In situ hybridization with probe for 18S/28S rDNA loci in *Abronia campbelli* (**a**), *Abronia deppii* (**b**,**c**), *Abronia graminea* (**d**), *Abronia lythrochila* (**e**,**f**), *Abronia mixteca* (**g**), *Barisia rudicollis* (**h**,**i**), *Celestus warreni* (**j**,**k**), *Gerrhonotus liocephalus* (**l**), *Heloderma exasperatum* (**m**,**n**) and *Heloderma horridum* (**o**,**p**).

**Figure 9:**
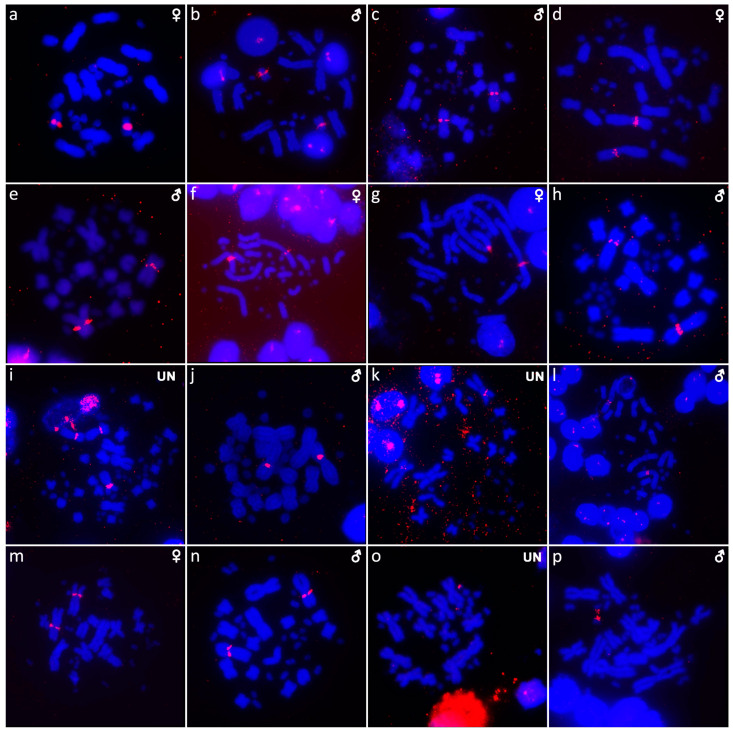
In situ hybridization with probe for 18S/28S rDNA loci in *Shinisaurus crocodilurus* (**a**,**b**), *Varanus auffenbergi* (**c**), *Varanus cumingi* (**d**,**e**), *Varanus kordensis* (**f**), *Varanus primordius* (**g**,**h**), *Varanus olivaceus* (**i**,**j**), *Varanus salvadorii* (**k**,**l**), *Varanus salvator komaini* (**m**,**n**), *Xenosaurus platyceps* (**o**) and *Xenosaurus rectocollaris* (**p**). UN: sex not known.

**Figure 10:**
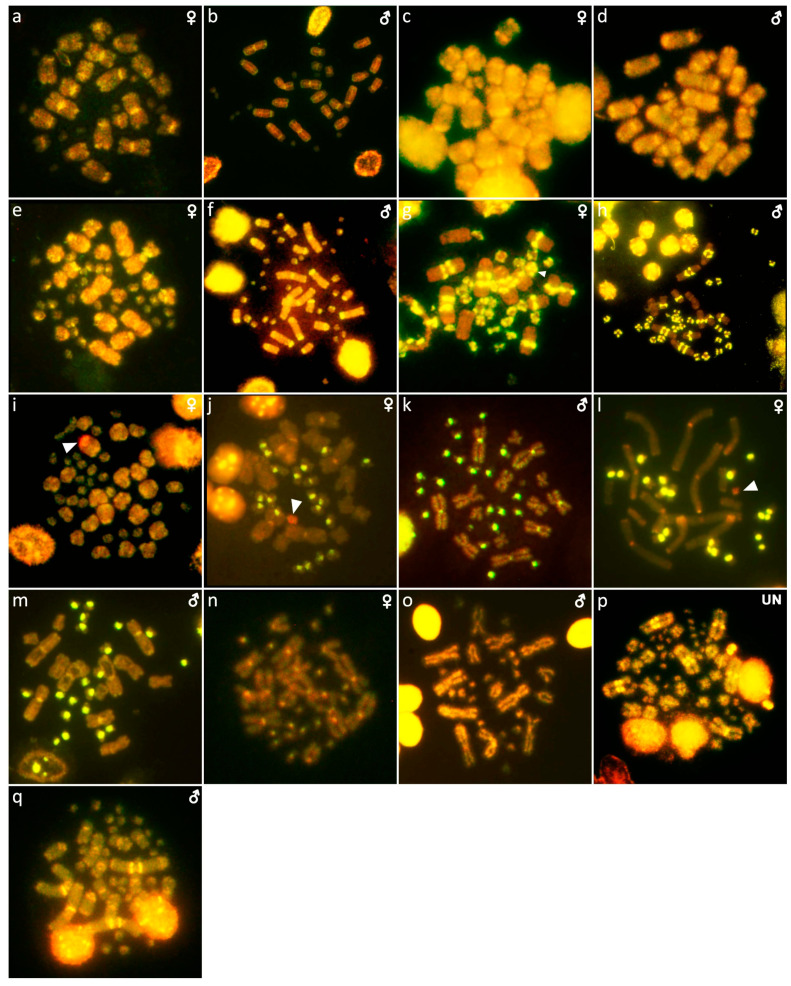
Comparative genome hybridization in *Abronia deppii* (**a**,**b**), *Abronia lythrochila* (**c**,**d**), *Barisia rudicollis* (**e**,**f**), *Celestus warreni* (**g**,**h**), *Gerrhonotus liocephalus* (**i**), *Heloderma exasperatum* (**j**,**k**), *Heloderma horridum* (**l**,**m**), *Shinisaurus crocodilurus* (**n**,**o**) and *Varanus primordius* (**p**,**q**). When visible, the W chromosomes are indicated by arrows. UN: sex not known.

Out of 23 species of anguimorphan lizards examined in the current study, 22 species have been to our knowledge cytogenetically studied for the first time here. We performed karyogram reconstruction from Giemsa-stained metaphases (Figure 1, Figure 2 and Figure 3), C-banding (Figure 4 and Figure 5), and FISH with probes for telomeric motifs (Figure 6 and Figure 7) and rDNA loci (Figure 8 and Figure 9) in all species, except *Abronia smithi, Abronia taeniata,* and *Xenosaurus grandis*, where only karyograms were prepared due to the small amount of the obtained chromosomal material (Figure 1 and Figure 3). In addition, CGH was performed in selected species (Figure 10).

### 3.1. Abronia campbelli *(Brodie & Savage, 1993)*

We examined only a male individual. Karyotype with 2n = 30 chromosomes (20 macro- and 10 microchromosomes). Among macrochromosomes, the pairs 1–3 and 7–10 are bi-armed, while the pairs 4–6 are acrocentric. The morphology of the microchromosomes cannot be determined (Figure 1a). The C-banding revealed heterochromatin mainly in centromeric regions of all macrochromosomes (Figure 4a). The telomeric sequences were detected only in the terminal positions of the chromosomes (Figure 6a). The FISH with rDNA probe revealed a signal on the 10th pair of macrochromosomes (Figure 8a).

### 3.2. Abronia deppii *(Wiegmann, 1828)*

Karyotype with 2n = 30 chromosomes (20 macro- and 10 microchromosomes). The pairs 1–3 and 7–10 are bi-armed, while the pairs 4–6 are acrocentric (Figure 1b,c). The C-banding revealed heterochromatin mainly in the centromeric regions of all macrochromosomes (Figure 4b,c). The telomeric sequences were detected only in the terminal positions of the chromosomes (Figure 6b,c). The FISH with rDNA probe revealed a signal on the 10th pair of macrochromosomes (Figure 8b,c). CGH did not detect any sex-specific differences (Figure 10a,b).

### 3.3. Abronia graminea *(Cope, 1864)*

We examined only a male individual. Karyotype with 2n = 30 chromosomes (20 macro- and 10 microchromosomes). The pairs 1–3, 8 and 10 are bi-armed, while the pairs 4–7 and 9 are acrocentric. Polymorphism was detected in the chromosomal pair 10, which consists of one submetacentric and one subtelocentric chromosome (Figure 1d). The C-banding revealed heterochromatin in the telomeric and centromeric regions of all macrochromosomes and in some microchromosomes (Figure 4d). The telomeric sequences were detected only in the terminal positions of the chromosomes (Figure 6d). The FISH with rDNA probe revealed a signal on the 10th pair of the karyogram (Figure 8d).

### 3.4. Abronia lythrochila *Smith & Alvarez del Toro, 1963*

Karyotype with 2n = 30 chromosomes (20 macro- and 10 microchromosomes). The pairs 1–3 and 7–10 are bi-armed, while the pairs 4–6 are acrocentric (Figure 1e,f). The C-banding revealed the accumulation of heterochromatin in the centromeric regions of all macrochromosomes and in the telomeric regions of some macrochromosomes (Figure 4e,f). A prominent accumulation was revealed on one of the microchromosomes in the female (Figure 4c). The telomeric sequences were detected only in the terminal positions of all chromosomes (Figure 6e,f). The FISH with rDNA probe revealed a signal on the 10th macrochromosome pair (Figure 7e,f). CGH did not detect any sex-specific differences (Figure 10c,d).

### 3.5. Abronia mixteca *Bogert & Porter, 1967*

We examined only a male individual. The karyotype with 2n = 30 chromosomes (20 macro- and 10 microchromosomes). The pairs 1–3 and 7–10 are bi-armed, while the pairs 4–6 are acrocentric (Figure 1g). The C-banding revealed an accumulation of heterochromatin in the centromeric regions of the macrochromosomes and in the telomeric region of a pair of macrochromosomes (Figure 4g). Some microchromosomes are also enriched in heterochromatin. The telomeric sequences were detected only in the terminal positions of all chromosomes (Figure 6g). The FISH with rDNA probe revealed a signal on the 10th pair of macrochromosomes (Figure 8g).

### 3.6. Abronia smithi *Campbell & Frost, 1993*

We examined only a male individual. Karyotype with 2n = 30 chromosomes (20 macro- and 10 microchromosomes). The chromosome pairs 1–3 and 7–10 are bi-armed, while the pairs 4–6 are acrocentric (Figure 1h).

### 3.7. Abronia taeniata *(Wiegmann, 1828)*

We examined only a male individual. Karyotype with 2n = 30 chromosomes (20 macro- and 10 microchromosomes). The chromosome pairs 1–3 and 7–10 are bi-armed, while the pairs 4–6 are acrocentric (Figure 1i).

### 3.8. Barisia rudicollis *(Wiegmann, 1828)*

Karyotype with 2n = 44 chromosomes (18 can be classified as macro- and 26 as microchromosomes, but the distinction is not prominent). The 1st and 2nd chromosome pairs are metacentric, the pairs 3–9 are acrocentric (Figure 2a,b). Heterochromatin is accumulated in the centromeric regions (Figure 4h,i). The telomeric sequences were detected only in the terminal positions (Figure 6h,i). The FISH with rDNA probe revealed a signal on a pair of microchromosomes (Figure 8h,i). CGH did not reveal any sex-specific differences (Figure 10e,f).

### 3.9. Celestus warreni *(Schwartz, 1970)*

Karyotype with 2n = 36 chromosomes (12 macro- and 14 microchromosomes). All macrochromosomes are bi-armed (Figure 2c,d). The C-banding revealed heterochromatin in the pericentromeric as well as telomeric regions of the macrochromosomes. The telomeric sequences were detected in the terminal positions of the chromosomes, in some of them also in the telomeric positions. A single microchromosome in the female possesses a notable accumulation of heterochromatin, which is not present in the male (Figure 4j,k). Interstitial telomeric repeats (ITRs) were detected in the pericentromeric regions of the chromosome pairs 1–5 (Figure 6j,k). The FISH with rDNA probe revealed a signal in the telomeric region of the 2nd chromosome pair (Figure 8j,k). CGH did not reveal any sex-specific differences (Figure 10g,h).

### 3.10. Gerrhonotus liocephalus *(Wiegmann, 1828)*

We examined only a female individual. Karyotype with 2n = 48 chromosomes (22 macro- and 26 microchromosomes, but the differences in size are more gradual). All macrochromosomes are acrocentric (Figure 2e). The C-banding revealed heterochromatic regions in the centromeric region of all chromosomes. Notably, a single chromosome from the first pair has a prominent accumulation of heterochromatin in comparison to other chromosomes (Figure 4l). The telomeric sequences were detected only in the terminal positions of all chromosomes (Figure 6l). The FISH with rDNA probe revealed a signal on one pair of microchromosomes (Figure 8l). Although the chromosomal material from the male individual was not available to us, we isolated DNA from a male, which we used for CGH. By using CGH method we detected a female-specific region in the female metaphases. The signal was detected in one of the chromosomes from the 1st chromosome pair (Figure 10i).

### 3.11. Heloderma exasperatum *Bogert & Martin del Campo, 1956*

Karyotype with 2n = 36 chromosomes (14 macro- and 22 microchromosomes). All macrochromosomes are bi-armed (Figure 2f,g). C-banding revealed heterochromatin mainly in the centromeric regions, in female animals the heterochromatin was accumulated also on the W chromosome (Figure 4m,n). ITRs were detected in 10 pairs of microchromosomes (Figure 6m,n). The FISH with rDNA probe revealed a signal at the telomeric region of the 3rd or 4th (both chromosome pairs have the same morphology) and the 5th chromosome pairs (Figure 8m,n). CGH revealed a female-specific signal in a single microchromosome in females, which corresponds to the W chromosome (Figure 10j,k).

### 3.12. Heloderma horridum *(Wiegmann, 1828)*

Karyotype with 2n = 36 chromosomes (14 macro- and 22 microchromosomes). All pairs of macrochromosomes are bi-armed (Figure 2h,i). The C-banding revealed heterochromatin in the centromeric region of all macrochromosomes and in the W chromosome of the females (Figure 4o,p). ITRs were detected in 10 pairs of microchromosomes (Figure 6o,p). The FISH with rDNA probe revealed a signal at the telomeric region of the 3rd or 4th (not possible to distinguish, as both chromosome pairs have the same morphology) and the 5th chromosome pairs (Figure 8o,p). CGH revealed a female-specific signal in a single microchromosome in the metaphases from the female specimen, which corresponds to the W chromosome (Figure 10l,m).

### 3.13. Shinisaurus crocodilurus *Ahl, 1930*

Karyotype with 2n = 32 chromosomes (14 macro- and 18 microchromosomes) (Figure 2j,k). The chromosome pairs 1–5 are bi-armed, while the pairs 6 and 7 are acrocentric. The C-banding revealed accumulation of heterochromatin in the centromeric region of the chromosomes, with a similar pattern in both sexes (Figure 5a,b). The telomeric sequences were detected only at the terminal positions of all chromosomes (Figure 7a,b). The FISH with rDNA probe revealed a signal at the pericentromeric region of the 6th chromosomal pair (Figure 9a,b). CGH method did not reveal any sex-specific differences (Figure 10n,o).

### 3.14. Varanus auffenbergi *Sprackland, 1999*

We examined only a male individual. Karyotype with 2n = 40 chromosomes (16 macro- and 24 microchromosomes). All macrochromosomes are bi-armed with the exception of the 4th chromosomal pair which is acrocentric (Figure 3a). The C-banding revealed the accumulation of heterochromatin in the pericentromeric and telomeric regions of the macrochromosomes (Figure 5c). The telomeric sequences were detected only in the terminal positions of all chromosomes (Figure 7c). FISH with rDNA probe revealed a signal in the pericentromeric region of the 1st chromosome pair (Figure 9c).

### 3.15. Varanus cumingi* Martin, 1893*

Karyotype with 2n = 40 chromosomes (16 macro- and 24 microchromosomes). The 4th chromosomal pair is acrocentric, while the other macrochromosomes are bi-armed (Figure 3b,c). The C-banding revealed the accumulation of heterochromatin in the pericentromeric region of all chromosomes and the telomeric region of the second chromosome pair. The W chromosomes in females are heterochromatic (Figure 5d,e). The telomeric sequences were detected only in the terminal positions of all chromosomes (Figure 7d,e). FISH with the rDNA probe revealed a signal in the pericentromeric region of the 1st chromosome pair (Figure 9d,e).

### 3.16. Varanus kordensis *(Meyer, 1874)*

We examined only a female individual. Karyotype with 2n = 40 chromosomes (16 macro- and 24 microchromosomes). All macrochromosomes are bi-armed except the 4th chromosome pair which is acrocentric (Figure 3d). The C-banding revealed accumulation of heterochromatin in the pericentromeric region of the chromosomes and in the W chromosome in females (Figure 5f). The telomeric sequences were detected only in the terminal positions of all chromosomes (Figure 7f). FISH with rDNA probe revealed a signal in the pericentromeric region of the 1st chromosome pair (Figure 9f).

### 3.17. Varanus olivaceus *Hallowell, 1857*

Karyotype with 2n = 40 chromosomes (16 macro- and 24 microchromosomes). All macrochromosomes are bi-armed, except the 4th pair which is acrocentric (Figure 3e,f). The C-banding revealed accumulation of heterochromatin mainly in the pericentromeric and telomeric regions of macrochromosomes and in the W chromosome in females (Figure 5g,h). The telomeric sequences were detected only in the terminal positions of all chromosomes (Figure 7g,h). The FISH with rDNA probe revealed a signal in the pericentromeric region of the 1st chromosome pair (Figure 9g,h).

### 3.18. Varanus primordius *Mertens, 1942*

We examined only a male individual. Karyotype with 2n = 40 chromosomes (16 macro- and 24 microchromosomes). The macrochromosome pairs 1–3 and 5–8 are bi-armed, the 4th pair is acrocentric (Figure 3h). An additional animal of unknown sex was identified as a triploid with 3n = 60 (Figure 2g). The C-banding revealed accumulation of heterochromatin mainly in the pericentromeric region of the chromosomes (Figure 5i,j). The telomeric sequences were detected only in the terminal positions of all chromosomes (Figure 7i,j). The FISH with rDNA probe revealed a signal in the pericentromeric region of the 1st chromosome pair (Figure 9i,j). The qPCR test revealed that the triploid individual had an average of 0.71 gene dose ratio for the Z-specific genes in comparison to the male, which corresponds to ZZW sex chromosome constitution (Table S2). The W chromosome cannot be accurately identified in the metaphases by the C-banding (Figure 5i,j) or CGH (Figure 10p,q), due to its small size and the bright signals on the other microchromosomes.

### 3.19. Varanus salvadorii *(Peters & Doria, 1878)*

Karyotype with 2n = 40 chromosomes (16 macro- and 24 microchromosomes). The chromosomal pairs 1–3 and 5–8 are bi-armed, while the pair 4 is acrocentric (Figure 3i,j). The C-banding revealed the accumulation of heterochromatin in the pericentromeric region of the chromosomes and in the W chromosome in females (Figure 5k,l). The telomeric sequences were detected in the terminal positions of all chromosomes. ITRs were detected in the pericentromeric region of the 6th chromosome pair (Figure 7k,l). The FISH with rDNA probe revealed a signal in the pericentromeric region of the 1st chromosome pair (Figure 9k,l).

### 3.20. Varanus salvator komaini *Nutaphand 1987*

Karyotype with 2n = 40 chromosomes (16 macro- and 24 microchromosomes). All macrochromosomes are bi-armed, except the 4th pair which is acrocentric (Figure 3k,l). The C-banding revealed accumulation of heterochromatin in the pericentromeric region of all chromosomes, in the telomeric region of the second chromosomal pair, and in the W of the female (Figure 5m,n). The telomeric sequences were detected only in the terminal positions of all chromosomes (Figure 7m,n). The FISH with rDNA probe revealed a signal in the pericentromeric region of the 1st chromosome pair (Figure 9m,n).

### 3.21. Xenosaurus grandis *(Gray, 1856)*

We examined only a single male individual. Karyotype with 2n = 36 chromosomes (12 macro- and 24 microchromosomes). All macrochromosomes are bi-armed (Figure 3m).

### 3.22. Xenosaurus platyceps *King & Thompson, 1968*

Two individuals of unknown sex were examined. Karyotype with 2n = 36 chromosomes (12 macro- and 24 microchromosomes). All macrochromosomes are bi-armed (Figure 3n). The C-banding revealed the accumulation of heterochromatin in the pericentromeric regions (Figure 5o). The telomeric sequences were detected in the terminal positions of all chromosomes and ITRs were detected on the 5th chromosomal pair (Figure 7o). The FISH with rDNA probe revealed a signal on one pair of microchromosomes (Figure 9o).

### 3.23. Xenosaurus rectocollaris *Smith & Iverson, 1993*

Only female individuals were examined. Karyotype with 2n = 36 chromosomes (12 macro- and 24 microchromosomes). All macrochromosomes are bi-armed (Figure 3o). C-banding revealed heterochromatin in the centromeric regions of all macrochromosomes and in some microchromosomes (Figure 5p). The telomeric sequences were detected only in the terminal positions of all chromosomes (Figure 7p). The FISH with rDNA probe revealed a signal on one pair of the microchromosomes (Figure 9p).

## 4. Discussion

**Figure 11:**
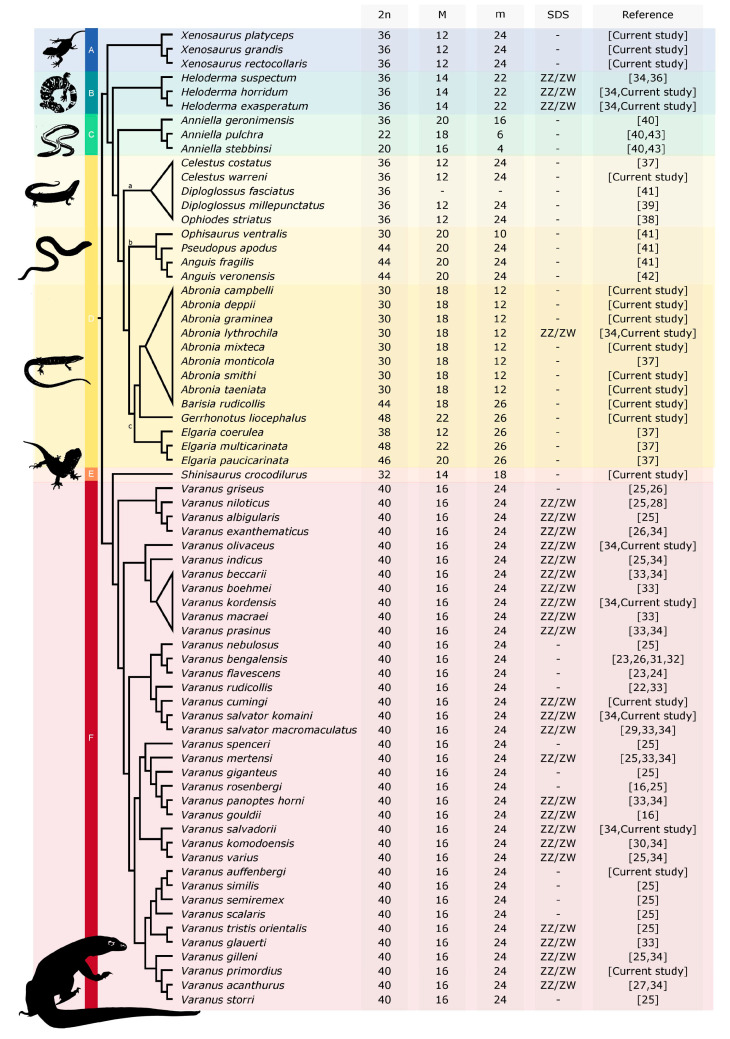
Evolution of karyotypes in anguimorphan reptiles. 2n: diploid chromosome number, M: number of macrochromosomes, m: number of microchromosomes, SDS: sex determination system. A: Xenosauridae, B: Helodermatidae, C: Anniellidae, D: Anguidae, E: Shinisauridae, F: Varanidae, a: Diploglossinae, b: Anguinae, c: Gerrhonotinae. The phylogenetic relationships follow [21,68,69,70,71].

The diploid chromosome numbers are in general variable in Anguimorpha (Figure 11), from 2n = 20 in *Anniella stebbinsi* (reported by Bezy et al. [40] as *Anniella pulchra*; [43]) to 2n = 48 in *Elgaria multicarinata* [37] and *Gerrhonotus liocephalus* (current study). However, the variability in chromosome number is highly unequally distributed among anguimorphan families, with most of the variability concentrated in the families Anguidae and Anniellidae. On the contrary, other anguimorphan lineages have much more stable chromosome numbers. Karyotypes with 2n = 40 chromosomes were identified in all 36 studied species of monitors (family Varanidae), 2n = 36 chromosomes in three examined species of helodermatids (family Helodermatidae), and in three species of the knob-scaled lizards (family Xenosauridae). The karyotype with 2n = 32 chromosomes was revealed in the current study in the only species of the family Shinisauridae (Figure 11).

Notably, 2n = 36 chromosomes is the most common diploid chromosome number in the species of the clade Toxicofera, and it is considered the ancestral state for caenophidian snakes [48], chameleons [49], and potentially also in iguanas and agamid lizards [29,50]. These estimations were largely based on the distribution of diploid chromosome number variation across the phylogenetic spectrum of each lineage, and at least in the case of snakes and iguanas, the reconstructed ancestral karyotypes differ in several interchromosomal rearrangements and thus in chromosome morphology. We think that the ancestral karyotype of the anguimorphan reptiles cannot be accurately reconstructed at the current state of knowledge, due to the extensive variability in diploid chromosome numbers and chromosome morphology. The karyotype evolution of the anguimorphan reptiles should be reconstructed in future studies either by comparative chromosome painting as applied in monitors [33] or by comparing chromosome level assemblies, which are currently available only for the Komodo dragon, *Varanus komodoensis* [35] and the Chinese crocodile lizard, *Shinisaurus crocodilurus* [51]. We are confident that the recent advances in next-generation sequencing technologies will make chromosome level assemblies feasible for additional phylogenetically informative species in the near future.

In addition to the terminal position (Figure 6 and Figure 7), interstitial telomeric-like repeats were detected in five out of 20 studied species, namely, in *Celestus warreni, Heloderma exasperatum, Heloderma horridum, Varanus salvadorii,* and *Xenosaurus platyceps*. In both species of the genus *Heloderma,* extensive amplification of telomeric repeats were identified in several pairs of microchromosomes. Telomeric repeats often tend to accumulate in the microchromosomes of sauropsids, as was documented in birds [52], turtles [44,53,54], and snakes [55]. Microchromosomes have generally higher rates of recombination in comparison to macrochromosomes [56,57,58]; therefore, we can speculate that telomeres extensively amplify in microchromosomes as a consequence of the DNA repair mechanism occurring after recombination events [59,60]. ITRs were detected in a single pair of macrochromosomes in *Xenosaurus platyceps* and *Varanus salvadorii*, and in five pairs of macrochromosomes in *Celestus warreni*. The origin of the ITRs is not clear, it might be a result of chromosomal rearrangements, such as chromosomal fusions or inversions, as well as activity of retrotransposons [61,62,63,64]. In addition, telomeric-like sequences are often part of the (peri)centromeric satellite motifs [65,66]. Overall, the presence of ITRs is quite rare in anguimorphan reptiles, in comparison to other toxicoferan reptiles, such as “haenophidian” snakes [67] and chameleons [49].

Animals often show variability in the chromosomal position and the amount of accumulation of rDNA loci among and within species, e.g. [72,73,74,75,76,77,78]. The rDNA loci were detected only in one pair of chromosomes in most of the studied anguimorphan species, but their topology varies from a pair of microchromosomes in four species (*Barisia rudicollis*, *Gerrhonotus liocephalus*, *Xenosaurus platyceps*, *Xenosaurus rectocollaris*) to a pair of macrochromosomes in the remaining studied species (Figure 8 and Figure 9). Notably, rDNA loci were detected in two pairs of macrochromosomes in *Heloderma horridum* and *H. exasperatum* (Figure 8). The presence of rDNA loci in multiple chromosome pairs is rather uncommon in reptiles, and it was previously reported in few species, such as the boa *Candoia paulsoni* [67] and the iguanas *Oplurus cyclurus* [74], *Leiocephalus carinatus* and *Leiocephalus raviceps* [72].

In addition, rDNA loci often accumulate on the sex chromosomes of reptiles. Notable differences in the copies of rDNA loci between the Z/W and X/Y sex chromosomes were previously reported in several species of trionychid and chelid turtles [53,79,80,81]. On the other hand, the rDNA loci are not detected on the Y chromosome of the common sandfish *Scincus scincus* (Scincidae) and the W chromosome of the Peters’ keeled plated lizard *Tracheloptychus petersi* (Gerrhosauridae), although they contain their X and Z chromosomes, respectively [7,82]. Notably, we did not detect any sex-specific accumulation on the sex chromosomes of anguimorphan reptiles (Figure 8 and Figure 9).

The chromosomal regions with rDNA loci tend to act as “hotspots” for recombination, which can explain, at least partially, the large variability in the accumulation and chromosome position of rDNA loci [73]. Such variability can be neutral and tolerated by the cell. In support, the comparison of silver-stained NORs (i.e., transcriptionally active rDNA loci) and rDNA-FISH (i.e., all chromosome positions with rDNA loci) revealed variability in the activity of rDNA loci [83,84,85]. Therefore, we speculate that the genome of some species might have more copies of rDNA loci than the minimum requirement for proper cellular function. For example, only 25–50% of rDNA loci are needed for normal development in the African clawed frog, *Xenopus laevis* [86]. On the other hand, the variability in rDNA loci can cause the development of cancer [87,88] and genome instability [89].

During the cytogenetic analysis, we identified a triploid individual of *Varanus primordius* (3n = 60) with the ZZW combination of sex chromosomes revealed by qPCR (Table S2). The sex of the individual was not possible to identify accurately by morphology. Another case of triploidy in the family Varanidae was previously reported in a male individual of *Varanus albigularis* with ZZZ sex chromosomes [33]. The spontaneous triploidy occurs rarely in reptilian species with a typical diploid karyotype. In addition to the above-mentioned cases of monitors, to the best of our knowledge, spontaneous triploidy was previously described in two species of snakes, *Elaphe bimaculata* [90] and *Agkistrodon piscivorus* [91]. Among reptiles, several species of obligatory parthenogens of hybrid origin are triploids [92,93,94,95,96,97,98,99,100]. The identification of spontaneous triploidy in adults and the existence of obligatory parthenogenetic triploid species show that triploid individuals are viable in many reptile lineages.

The C-banding revealed heterochromatin typically in the (peri)centromeric regions, and occasionally in the telomeric regions (Figure 6 and Figure 7). The W chromosomes were detected by C-banding, in all studied species of helodermatids and monitors, except *V. primordius* (Figure 4, Figure 5 and Figure 10). Putative W chromosomes were detected in *Abronia lythrochila*, *Celestus warreni,* and *Gerrhonotus liocephalus*. In *Abronia lythrochila,* the C-banding (Figure 4e,f) revealed a stronger accumulation of the heterochromatin in a single microchromosome, which agrees with the previous report of a ZZ/ZW system in this species [34]; however, no sex-specific differences were detected by CGH (Figure 10c,d). Notably, C-banding and CGH did not reveal any sex-specific pattern in the closely related species *Abronia deppii* (Figure 4b,c and Figure 10a,b), which indicates that the species of the genus *Abronia* might not share homologous, or at least not equally differentiated sex chromosomes. In *Celestus warreni*, female-specific signal was detected in a single microchromosome by both C-banding (Figure 4j,k) and CGH (Figure 10g,h). In *Gerrhonotus liocephalus*, female-specific signal was detected in a single chromosome from the 1st pair by both C-banding (Figure 4l) and CGH (Figure 10i). However, chromosomal material was not available from a male in *Gerrhonotus liocephalus*, and we were thus not able to compare both sexes to validate the presence of a ZZ/ZW sex determination system.

We assume that sex chromosomes are likely homomorphic and poorly differentiated in the rest of the studied anguimorphan species, although we cannot exclude that perhaps some of these species might have environmental sex determination (ESD). To expand our knowledge on sex determination and sex chromosome evolution in the anguimorphan lizards, we will need to use advanced genomic/bioinformatic analysis (such as RADseq, genome coverage analysis), as well as incubation experiments for the detection of possible ESD.

## Data Availability

All data are provided in the current manuscript.

## Supplementary Materials

The following are available online at https://www.mdpi.com/article/10.3390/cells10071612/s1, Table S1: Summary of studied species and number of examined individuals, Table S2: Primers and results of the qPCR test for estimating the sex chromosome constitution in the triploid *Varanus primordius*.

## References

[1] Wilson M.A., Makova K.D. (2009). Genomic analyses of sex chromosome evolution. Rev. Genom. Hum. Genet..

[2] Bachtrog D., Mank J.E., Peichel C.L., Kirkpatrick M., Otto S.P., Ashman T.L., Hahn M.W., Kitano J., Mayrose I., Ming R. (2014). Tree of sex consortium. Sex determination: Why so many ways of doing it?. PLoS Biol..

[3] Pennell M.W., Mank J.E., Peichel C.L. (2018). Transitions in sex determination and sex chromosomes across vertebrate species. Mol. Ecol..

[4] Ohno S. (1967). Sex Chromosomes and Sex-Linked Genes.

[5] Rice W.R. (1987). The accumulation of sexually antagonistic genes as a selective agent promoting the evolution of reduced recombination between primitive sex chromosomes. Evolution.

[6] Charlesworth B. (1996). The evolution of chromosomal sex determination and dosage compensation. Curr. Biol..

[7] Kostmann A., Kratochvíl L., Rovatsos M. (2021). Poorly differentiated XX/XY sex chromosomes are widely shared across skink radiation. Proc. R. Soc. B.

[8] Rovatsos M., Johnson Pokorná M., Altmanová M., Kratochvíl L. (2015). Female heterogamety in Madagascar chameleons (Squamata: Chamaeleonidae: *Furcifer*): Differentiation of sex and neo-sex chromosomes. Sci. Rep..

[9] Rovatsos M., Altmanová M., Augstenová B., Mazzoleni S., Velenský P., Kratochvíl L. (2019). ZZ/ZW sex determination with multiple neo-sex chromosomes is common in Madagascan chameleons of the genus *Furcifer* (Reptilia: Chamaeleonidae). Genes.

[10] Epplen J.T., McCarrey J.R., Sutou S., Ohno S. (1982). Base sequence of a cloned snake W-chromosome 65DNA fragment and identification of a male-specific putative mRNA in the mouse. Proc. Natl. Acad. Sci. USA.

[11] Nanda I., Deubelbeiss C., Guttenbach M., Epplen J.T., Schmid M. (1990). Heterogeneities in the distribution of (GACA)_n_ simple repeats in the karyotypes of primates and mouse. Hum. Genet..

[12] Steinemann S., Steinemann M. (2005). Retroelements: Tools for sex chromosome evolution. Cytogenet. Genome Res..

[13] Kejnovsky E., Hobza R., Cermak T., Kubat Z., Vyskot B. (2009). The role of repetitive DNA in structure and evolution of sex chromosomes in plants. Heredity.

[14] O’Meally D., Patel H.R., Stiglec R., Sarre S.D., Georges A., Marshall Graves J.A., Ezaz T. (2010). Non-homologous sex chromosomes of birds and snakes share repetitive sequences. Chromosome Res..

[15] Pokorná M., Kratochvíl L., Kejnovský E. (2011). Microsatellite distribution on sex chromosomes at different stages of heteromorphism and heterochromatinization in two lizard species (Squamata: Eublepharidae: *Coleonyx elegans* and Lacertidae: *Eremias velox*). BMC Genet..

[16] Matsubara K., Sarre S.D., Georges A., Matsuda Y., Graves J.A.M., Ezaz T. (2014). Highly differentiated ZW sex microchromosomes in the Australian *Varanus* species evolved through rapid amplification of repetitive sequences. PLoS ONE.

[17] Matsubara K., O’Meally D., Azad B., Georges A., Sarre S.D., Graves J.A.M., Matsuda Y., Ezaz T. (2016). Amplification of microsatellite repeat motifs is associated with the evolutionary differentiation and heterochromatinization of sex chromosomes in Sauropsida. Chromosoma.

[18] Augstenová B., Mazzoleni S., Kratochvíl L., Rovatsos M. (2018). Evolutionary dynamics of the W chromosome in caenophidian snakes. Genes.

[19] Suwala G., Altmanová M., Mazzoleni S., Karameta E., Pafilis P., Kratochvíl L., Rovatsos M. (2020). Evolutionary variability of W-linked repetitive content in Lacertid lizards. Genes.

[20] Uetz P., Freed P., Hošek J. (2020). The Reptile Database. http://www.reptile-database.org.

[21] Pyron R.A., Burbrink F.T., Wiens J.J. (2013). A phylogeny and revised classification of Squamata, including 4161 species of lizards and snakes. BMC Evol. Biol..

[22] Gorman G.C., Gress F. (1970). Chromosome cytology of four boid snakes and a varanid lizard, with comments on the cytosystematics of primitive snakes. Herpetologica.

[23] Singh L., Sharma T., Ray-Chaudhu S.P. (1970). Chromosome numbers and sex chromosomes in few Indian species of amphibia and reptiles. Mamm. Chrom. News.

[24] Singh L. (1974). Study of mitotic and meiotic chromosomes in seven species of lizards. Proc. Zool. Soc..

[25] King M., King D. (1975). Chromosomal evolution in the lizard genus *Varanus* (Reptilia). Aust. J. Biol. Sci..

[26] De Smet W.H.O. (1981). Description of the orsein stained karyotypes of 136 lizard species (Lacertilia, Reptilia) belonging to the families Teiidae, Scincidae, Lacertidae, Cordylidae and Varanidae (Autarchoglossa). Acta Zool. Pathol. Antverp..

[27] King M., Mengden G.A., King D. (1982). A pericentric-inversion polymorphism and a ZZ/ZW sex-chromosome system in *Varanus acanthurus* Boulenger analysed by G- and C-banding and Ag staining. Genetica.

[28] Porter C., Haiduk M., De Queiroz K. (1994). Evolution and phylogenetic significance of ribosomal gene location in chromosomes of squamate reptiles. Copeia.

[29] Srikulnath K., Uno Y., Nishida C., Matsuda Y. (2013). Karyotype evolution in monitor lizards: Cross-species chromosome mapping of cDNA reveals highly conserved synteny and gene order in the Toxicofera clade. Chromosome Res..

[30] Johnson Pokorná M., Altmanová M., Rovatsos M., Velenský P., Vodička R., Rehák I., Kratochvíl L. (2016). First description of the karyotype and sex chromosomes in the Komodo dragon (*Varanus komodoensis*). Cytogenet. Genome Res..

[31] Patawang I., Tanomtong A. (2017). Constitutive heterochromatin observed on metaphase chromosome of *Varanus bengalensis* by C-banding and DAPI methods. Cytologia.

[32] Patawang I., Tanomtong A., Getlekha N., Phimphan S., Pinthong K., Neeratanaphan L. (2017). Standardized karyotype and idiogram of bengal monitor lizard, Varanus bengalensis (Squamata, Varanidae). Cytologia.

[33] Iannucci A., Altmanová M., Ciofi C., Ferguson-Smith M., Milan M., Pereira J.C., Pether J., Rehák I., Rovatsos M., Stanyon R. (2019). Conserved sex chromosomes and karyotype evolution in monitor lizards (Varanidae). Heredity.

[34] Rovatsos M., Rehák I., Velenský P., Kratochvíl L. (2019). Shared ancient sex chromosomes in varanids, beaded lizards, and alligator lizards. Mol. Biol. Evol..

[35] Lind A.L., Lai Y.Y., Mostovoy Y., Holloway A.K., Iannucci A., Mak A.C.Y., Fondi M., Orlandini V., Eckalbar W.L., Milan M. (2019). Genome of the Komodo dragon reveals adaptations in the cardiovascular and chemosensory systems of monitor lizards. Nat. Ecol. Evol..

[36] Johnson Pokorná M., Rovatsos M., Kratochvíl L. (2014). Sex chromosomes and karyotype of the (nearly) mythical creature, the Gila monster*, Heloderma suspectum* (Squamata: Helodermatidae). PLoS ONE.

[37] Bury R.B., Gorman G.C., Lynch J.F. (1969). Karyotypic data for five species of anguid lizards. Experientia.

[38] Beçak M.L., Beçak W., Denaro L. (1972). Chromosome polymorphism, geographical variation and karyotypes in Sauria. Caryologia.

[39] Stamm B., Gorman G.C., Graham J.B. (1975). Notes on the chromosomes of Anolis agassizi (Sauria: Iguanidae) and Diploglossus millepunctatus (Sauria: Anguidae). The Biological Investigation of Malpelo Island.

[40] Bezy R.L., Gorman G.C., Kim Y.J., Wright J.W. (1977). Chromosomal and genetic divergence in the fossorial lizards of the family Anniellidae. Syst. Biol..

[41] Olmo E., Signorino G.G. (2005). Chromorep: A Reptile Chromosomes Database. http://chromorep.univpm.it.

[42] Mezzasalma M., Guarino F.M., Aprea G., Petraccioli A., Crottini A., Odierna G. (2013). Karyological evidence for diversification of Italian slow worm populations (Squamata, Anguidae). Comp. Cytogenet..

[43] Papenfuss T.J., Parham J.F. (2013). Four new species of California legless lizards (Anniella). Breviora.

[44] Mazzoleni S., Augstenová B., Clemente L., Auer M., Fritz U., Praschag P., Protiva T., Velenský P., Kratochvíl L., Rovatsos M. (2019). Turtles of the genera Geoemyda and Pangshura (Testudines: Geoemydidae) lack differentiated sex chromosomes: The end of a 40-year error cascade for Pangshura. PeerJ.

[45] Sumner A.T. (1972). A simple technique for demonstrating centromeric heterochromatin. Exp. Cell Res..

[46] Ijdo J.W., Baldini A., Ward D.C., Reeders S.T., Wells R.A. (1991). Origin of human chromosome 2: An ancestral telomere-telomere fusion. Proc. Natl. Acad. Sci. USA.

[47] Endow S.A. (1982). Polytenization of the ribosomal genes on the X and Y chromosomes of Drosophila melanogaster. Genetics.

[48] Oguiura N., Ferrarezzi H., Batistic R.F. (2009). Cytogenetics and molecular data in snakes: A phylogenetic approach. Cytogenet. Genome Res..

[49] Rovatsos M., Altmanová M., Johnson Pokorná M., Velenský P., Sanchez Baca A., Kratochvíl L. (2017). Evolution of karyotypes in chameleons. Genes.

[50] Gorman G., Chiarelli A.B., Capanna E. (1973). The Chromosomes of the Reptilia, a Cytotaxonomic Interpretation. Cytotaxonomy and Vertebrate Evolution.

[51] Gao J., Li Q., Wang Z., Zhou Y., Martelli P., Li F., Xiong Z., Wang J., Yang H., Zhang G. (2017). Sequencing, de novo assembling, and annotating the genome of the endangered Chinese crocodile lizard Shinisaurus crocodilurus. GigaScience.

[52] Nanda I., Schrama D., Feichtinger W., Haaf T., Schartl M., Schmid M. (2002). Distribution of telomeric (TTAGGG)_n_ sequences in avian chromosomes. Chromosoma.

[53] Mazzoleni S., Augstenová B., Clemente L., Auer M., Fritz U., Praschag P., Protiva T., Velenský P., Kratochvíl L., Rovatsos M. (2020). Sex is determined by XX/XY sex chromosomes in Australasian side-necked turtles (Testudines: Chelidae). Sci. Rep..

[54] Clemente L., Mazzoleni S., Pensabene Bellavia E., Augstenová B., Auer M., Praschag P., Protiva T., Velenský P., Wagner P., Fritz U. (2020). Interstitial telomeric repeats are rare in turtles. Genes.

[55] Rovatsos M., Pokorná M.J., Kratochvíl L. (2015). Differentiation of sex chromosomes and karyotype characterisation in the dragon snake *Xenodermus javanicus* (Squamata: Xenodermatidae). Cytogenet. Genome Res..

[56] Burt D.W. (2002). Origin and evolution of avian microchromosomes. Cytogenet. Genome Res..

[57] International Chicken Genome Sequencing Consortium (2004). Sequence and comparative analysis of the chicken genome provide unique perspectives on vertebrate evolution. Nature.

[58] Backström N., Forstmeier W., Schielzeth H., Mellenius H., Nam K., Bolund E., Webster M.T., Öst T., Schneider M., Kempenaers B. (2010). The recombination landscape of the zebra finch *Taeniopygia guttata* genome. Genome Res..

[59] Melek M., Shippen D.E. (1996). Chromosome healing: Spontaneous and programmed de novo telomere formation by telomerase. Bioessays.

[60] Shay J.R., Wright W.E. (2019). Telomeres and telomerase: Three decades of progress. Nat. Rev. Genet..

[61] Bolzán A.D. (2017). Interstitial telomeric sequences in vertebrate chromosomes: Origin, function, instability and evolution. Mutat. Res..

[62] Bolzán A.D., Bianchi M.S. (2006). Telomeres, interstitial telomeric repeat sequences, and chromosomal aberrations. Mutat. Res..

[63] Ruiz-Herrera A., Nergadze S.G., Santagostino M., Giulotto E. (2008). Telomeric repeats far from the ends: Mechanisms of origin and role in evolution. Cytogenet. Genome Res..

[64] Birchler J.A., Presting G.G. (2012). Retrotransposon insertion targeting: A mechanism for homogenization of centromere sequences on nonhomologous chromosomes. Genes. Dev..

[65] Rovatsos M.T., Marchal J.A., Romero-Fernández I., Fernández F.J., Giagia-Athanosopoulou E.B., Sánchez A. (2011). Rapid, independent, and extensive amplification of telomeric repeats in pericen-tromeric regions in karyotypes of arvicoline rodents. Chromosome Res..

[66] Rovatsos M., Marchal J.A., Giagia-Athanasopoulou E., Sánchez A. (2021). Molecular composition of heterochromatin and its contribution to chromosome variation in *Microtus thomasi/Microtus atticus* species complex. Genes.

[67] Augstenová B., Mazzoleni S., Kostmann A., Altmanová M., Frynta D., Kratochvíl L., Rovatsos M. (2019). Cytogenetic analysis did not reveal differentiated sex chromosomes in ten species of boas and pythons (Reptilia: Serpentes). Genes.

[68] Gvoždík V., Benkovský N., Crottini A., Bellati A., Moravec J., Romano A., Sacchi R., Jandzik D. (2013). An ancient lineage of slow worms, genus *Anguis* (Squamata: Anguidae), survived in the Italian Peninsula. Mol. Phylogenet. Evol..

[69] Lin L.-H., Wiens J.J. (2017). Comparing macroecological patterns across continents: Evolution of climatic niche breadth in varanid lizards. Ecography.

[70] De Oca A.N.M., Barley A.J., Meza-Lázaro R.N., García-Vázquez U.O., Zamora-Abrego J.G., Thomson R.C., Leaché A.D. (2017). Phylogenomics and species delimitation in the knob-scaled lizards of the genus *Xenosaurus* (Squamata: Xenosauridae) using ddRADseq data reveal a substantial underestimation of diversity. Mol. Phylogenet. Evol..

[71] Brennan I.G., Lemmon A.R., Lemmon E.M., Portik D.M., Weijola V., Welton L., Donnellan S.C., Keogh J.S. (2021). Phylogenomics of monitor lizards and the role of competition in dictating body size disparity. Syst. Biol..

[72] Porter C.A., Hamilton M.J., Sites J.W., Baker R.J. (1991). Location of ribosomal DNA in chromosomes of squamate reptiles: Systematic and evolutionary implications. Herpetologica.

[73] Stults D.M., Killen M.W., Pierce H.H., Pierce A.J. (2008). Genomic architecture and inheritance of human ribosomal RNA gene clusters. Genome Res..

[74] Altmanová M., Rovatsos M., Kratochvíl L., Johnson Pokorná M. (2016). Minute Y chromosomes and karyotype evolution in Madagascan iguanas (Squamata: Iguania: Opluridae). Biol. J. Linn. Soc..

[75] Sochorová J., Garcia S., Gálvez F., Symonová R., Kovařík A. (2017). Evolutionary trends in animal ribosomal DNA loci: Introduction to a new online database. Chromosoma.

[76] Mazzoleni S., Rovatsos M., Schillaci O., Dumas F. (2018). Evolutionary insight on localization of 18S, 28S rDNA genes on homologous chromosomes in Primates genomes. Comp. Cytogenet..

[77] Micolino R., Cristiano M.P., Travenzoli N.M., Lopes D.M., Cardoso D.C. (2019). Chromosomal dynamics in space and time: Evolutionary history of *Mycetophylax* ants across past climatic changes in the Brazilian Atlantic coast. Sci. Rep..

[78] Degrandi T.M., Gunski R.J., Garnero A.D.V., Oliveira E.H.C., Kretschmer R., Souza M.S. (2020). Barcellos, S.A.; Hass, I. The distribution of 45S rDNA sites in bird chromosomes suggests multiple evolutionary histories. Genet. Mol. Biol..

[79] Literman R., Badenhorst D., Valenzuela N. (2014). qPCR-based molecular sexing by copy number variation in r RNA genes and its utility for sex identification in soft-shell turtles. Methods Ecol. Evol..

[80] Literman R., Radhakrishnan S., Tamplin J., Burke R., Dresser C., Valenzuela N. (2017). Development of sexing primers in *Glyptemys insculpta* and *Apalone spinifera* turtles uncovers an XX/XY sex-determining system in the critically-endangered bog turtle *Glyptemys muhlenbergii*. Conserv. Genet. Resour..

[81] Rovatsos M., Praschag P., Fritz U., Kratochvíl L. (2017). Stable Cretaceous sex chromosomes enable molecular sexing in softshell turtles (Testudines: Trionychidae). Sci. Rep..

[82] Kostmann A., Kratochvíl L., Rovatsos M. (2021). First report of sex chromosomes in plated lizards (Squamata: Gerrhosauridae). Sex. Dev..

[83] Zurita F., Sánchez A., Burgos M., Jiménez R., de la Guardia R.D. (1997). Interchromosomal, intercellular and interindividual variability of NORs studied with silver staining and *in situ* hybridization. Heredity.

[84] Zurita F., Jimenez R., Burgos M., de La Guardia R.D. (1998). Sequential silver staining and *in situ* hybridization reveal a direct association between rDNA levels and the expression of homologous nucleolar organizing regions: A hypothesis for NOR structure and function. J. Cell Sci..

[85] Nirchio M., Oliveira C., Ferreira I.A., Granado A., Ron E. (2007). Extensive polymorphism and chromosomal characteristics of ribosomal DNA in the characid fish *Triportheus venezuelensis* (Characiformes, Characidae). Genet. Mol. Biol..

[86] Miller L., Knowland J. (1970). Reduction of ribosomal RNA synthesis and ribosomal RNA genes in a mutant of *Xenopus laevis* which organizes only a partial nucleolus: II. The number of ribosomal RNA genes in animals of different nucleolar types. J. Mol. Biol..

[87] Gibbons J.G., Branco A.T., Godinho S.A., Yu S., Lemos B. (2015). Concerted copy number variation balances ribosomal DNA dosage in human and mouse genomes. Proc. Natl. Acad. Sci. USA.

[88] Xu B., Li H., Perry J.M., Singh V.P., Unruh J., Yu Z., Zakari M., McDowell W., Li L., Gerton J.L. (2017). Ribosomal DNA copy number loss and sequence variation in cancer. PLoS Genet..

[89] Kobayashi T. (2011). How does genome instability affect lifespan? Roles of rDNA and telomeres. Genes Cells.

[90] Rovatsos M., Augstenová B., Altmanová M., Sloboda M., Kodym P., Kratochvíl L. (2018). Triploid colubrid snake provides insight into the mechanism of sex determination in advanced snakes. Sex. Dev..

[91] Tiersch T.R., Figiel C.R. (1991). A triploid snake. Copeia.

[92] Peters G. (1971). Die intragenerischen Gruppen und die Phylogenese der Schmetterlingsagamen (Agamidae: *Leiolepis)*. Zool. Jahrb. Syst..

[93] Moritz C. (1983). Parthenogenesis in the endemic Australian lizard *Heteronotia binoei* (Gekkonidae). Science.

[94] Moritz C., Case T.J., Bolger D.T., Donnellan S. (1993). Genetic diversity and the history of pacific island house geckos (*Hemidactylus* and *Lepidodactylus*). Biol. J. Linn. Soc..

[95] Darevsky I.S., Kupriyanova L.A., Roshchin V.V. (1984). A new all-female triploid species of gecko and karyological data on the bisexual *Hemidactylus frenatus* from Vietnam. J. Herpetol..

[96] Wynn A.H., Cole C.J., Gardner A.L. (1987). Apparent triploidy in the unisexual Brahminy blind snake, *Ramphotyphlops braminus*. Am. Mus. Novit..

[97] Adams M., Foster R., Hutchinson M.N., Hutchinson R.G., Donnellan S.C. (2003). The Australian scincid lizard Menetia greyii: A new instance of widespread vertebrate parthenogenesis. Evolution.

[98] Lutes A.A., Baumann D.P., Neaves W.B., Baumann P. (2011). Laboratory synthesis of an independently reproducing vertebrate species. Proc. Natl. Acad. Sci. USA.

[99] Vergun A.A., Martirosyan I.A., Semyenova S.K., Omelchenko A.V., Petrosyan V.G., Lazebny O.E., Tokarskaya O.N., Korchagin V.I., Ryskov A.P. (2014). Clonal diversity and clone formation in the parthenogenetic Caucasian rock lizard Darevskia dahli. PLoS ONE.

[100] Trifonov V.A., Paoletti A., Caputo Barucchi V., Kalinina T., O’Brien P.C.M., Ferguson-Smith M.A., Giovannotti M. (2015). Comparative chromosome painting and NOR distribution suggest a complex hybrid origin of triploid *Lepidodactylus lugubris* (Gekkonidae). PLoS ONE.

