# Supplementary material for: Cytogenetic Evidence for Sex Chromosomes and Karyotype Evolution in Anguimorphan Lizards
Source: Cells. 2021 Jun 28;10(7):1612. doi: 10.3390/cells10071612 (PMC8304200; doi:10.3390/cells10071612)
Supplement: Supplementary file 1 [file cells-10-01612-s001.zip › cells-1266014-Supplementary.pdf]

Cytogenetic Evidence for Sex Chromosomes and Karyotype Evolution in Aguimorphan Lizards

Barbora Augstenová, Eleonora Pensabene, Lukáš Kratochvíl and Michail Rovatsos *

Department of Ecology, Faculty of Science, Charles University, 12844 Prague, Czech Republic; augstenova.barbora@gmail.com (B.A.); pensabee@natur.cuni.cz (E.P.); kratoch1@natur.cuni.cz (L.K.)
* Correspondence: mirovatsos@gmail.com

Supplementary materials:

Table S1. Summary of studied species and number of examined individuals.

Family	Species	Male	Female	Unknown sex
Anguidae	*Abronia campbelli*	1	0	0
Anguidae	*Abronia dippei*	1	1	0
Anguidae	*Abronia graminea*	1	0	0
Anguidae	*Abronia lythrochila*	2	2	0
Anguidae	*Abronia mixteca*	1	0	0
Anguidae	*Abronia smithi*	1	0	0
Anguidae	*Abronia teaniata*	1	0	0
Anguidae	*Barisia rudicolis*	1	1	0
Anguidae	*Celestus warreni*	1	1	0
Anguidae	*Gerhonotus liocepfalus*	1	0	0
Helodermatidae	*Heloderma exasperatum*	3	2	0
Helodermatidae	*Heloderma horridum*	3	2	0
Shinisauridae	*Shinisaurus crocodilurus*	1	1	2
Varanidae	*Varanus auffenbergi*	1	0	0
Varanidae	*Varanus cumingi*	1	2	0
Varanidae	*Varanus komaini*	1	1	0
Varanidae	*Varanus kordiensis*	0	1	0
Varanidae	*Varanus olivaceus*	3	2	0
Varanidae	*Varanus primordius*	1	0	1
Varanidae	*Varanus salvadori*	1	1	0
Xenosauridae	*Xenosaurus grandis*	1	0	0
Xenosauridae	*Xenosaurus platyceps*	0	0	2
Xenosauridae	*Xenosaurus rectocollaris*	0	2	0

Table S2. Primers and results of the qPCR test for estimating the sex chromosome constitution in the triploid *Varanus primordius*.

Gene name	Gene symbol	Primer name	Forward primer	Reverse primer	triploid to male gene dose ratio
MDS1 and EVI1 complex locus	*mecom*	mecom_5	AGGAGATTTTGTGAGGGCAAGA	GCTGTTGGAAAGGTAAGACCAG	1.00
double-stranded RNA-specific editase B2	*adarb2*	adarb2_1	CTGCTGGGAATGCGACTGG	GCCTTTCGGAGACTGTGGAG	0.97
glutamate receptor ionotropic, NMDA 3B	*grin3b*	grin3b_1	ATGGCGTAGCAGAGGTTGAG	GTGGGTGGGCATCTTCGTG	0.71
